# NLRP3 inflammasome activation and pyroptosis are dispensable for tau pathology

**DOI:** 10.3389/fnagi.2024.1459134

**Published:** 2024-09-24

**Authors:** Ine Paesmans, Kristof Van Kolen, Marc Vandermeeren, Pei-Yu Shih, Dirk Wuyts, Fleur Boone, Sergio Garcia Sanchez, Karolien Grauwen, Filip Van Hauwermeiren, Nina Van Opdenbosch, Mohamed Lamkanfi, Geert van Loo, Astrid Bottelbergs

**Affiliations:** ^1^Janssen Research and Development, Janssen Pharmaceutica NV, Johnson & Johnson Company, Beerse, Belgium; ^2^Neuroscience Therapeutic Area, Janssen Research and Development, Beerse, Belgium; ^3^VIB Center for Inflammation Research, Ghent, Belgium; ^4^Department of Biomedical Molecular Biology, Ghent University, Ghent, Belgium; ^5^Department of Internal Medicine and Pediatrics, Ghent University, Ghent, Belgium

**Keywords:** *NLRP3*, *GSDMD*, P301S, OSCs, neurodegeneration and tau pathology

## Abstract

**Background:**

Neuroinflammation is widely recognized as a key factor in the pathogenesis of Alzheimer’s disease (AD), alongside ß-amyloid deposition and the formation of neurofibrillary tangles. The NLR family pyrin domain containing 3 (NLRP3) inflammasome, part of the innate immune system, has been implicated in the neuropathology of both preclinical amyloid and tau transgenic models. Activation of the NLRP3 pathway involves an initial priming step, which increases the expression of Nlrp3 and interleukin (IL)-1β, followed by the assembly of the NLRP3 inflammasome complex, comprising NLRP3, ASC, and caspase-1. This assembly leads to the proteolytic maturation of the pro-inflammatory cytokines IL-1β and IL-18. Additionally, the NLRP3 inflammasome induces Gasdermin D (GSDMD) cleavage, forming membrane pores through which IL-1β and IL-18 are secreted. Inhibition of NLRP3 has been shown to enhance plaque clearance by modulating microglial activation. Furthermore, blocking NLRP3 in tau transgenic mice has been found to reduce tau phosphorylation by affecting the activity of certain tau kinases and phosphatases.

**Methods:**

In this study, organotypic brain slice cultures from P301S transgenic mice were treated with lipopolysaccharide (LPS) plus nigericin as a positive control or exposed to tau seeds (K18) to evaluate NLRP3 inflammasome activation. The effect of tau seeding on NLRP3 activity was further examined using Meso Scale Discovery (MSD) assays to measure IL1β secretion levels in the presence and absence of NLRP3 inhibitors. The role of NLRP3 activity was investigated in full-body *Nlrp3* knockout mice crossbred with the tau transgenic P301S model. Additionally, full-body and microglia-selective *Gsdmd* knockout mice were crossbred with P301S mice, and tau pathology and neurodegeneration were evaluated at early and late stages of the disease using immunohistochemistry and biochemical assays.

**Results:**

Activation of the NLRP3 pathway was observed in the mouse organotypic slice culture (OSC) model following stimulation with LPS and nigericin or exposure to tau seeds. However, *Nlrp3* deficiency did not mitigate tauopathy or neurodegeneration in P301S mice *in vivo*, showing only a minor effect on plasma neurofilament (NF-L) levels. Consistently, *Gsdmd* deficiency did not alter tau pathology in P301S mice. Furthermore, neither full-body nor microglia-selective *Gsdmd* deletion had an impact on neuronal pathology or the release of pro-inflammatory cytokines.

**Conclusion:**

The absence of key components of the NLRP3 inflammasome pathway did not yield a beneficial effect on tau pathology or neurodegeneration in the preclinical Tau-P301S mouse model of AD. Nonetheless, organotypic slice cultures could serve as a valuable *ex vivo* mechanistic model for evaluating NLRP3 pathway activation and pharmacological inhibitors.

## Introduction

The key pathological features of Alzheimer’s disease (AD) include extracellular amyloid *β* (Aβ) plaque deposits and intraneuronal neurofibrillary tangles (NFTs), primarily composed of hyperphosphorylated tau, leading to neurodegeneration. Additionally, AD is consistently marked by a significant neuroinflammatory response, which is increasingly recognized as an important modulator of the disease’s pathogenic process (Heneka et al., 2015; Heppner et al., 2015). Inflammatory cells within the central nervous system (CNS), including microglia and astrocytes, cluster around amyloid deposits in postmortem AD brains (Arends et al., 2000; Beach et al., 1989). Positron emission tomography (PET) imaging with radioligands that bind to translocator protein TSPO on activated microglia has enabled *in vivo* imaging of microgliosis and revealed increased retention of these tracers in human AD brains, even in patients with mild dementia, indicating that microglia activation is an early event in the disease (Cagnin et al., 2001; Okello et al., 2009).

Neuroinflammation is primarily an innate immune response with microglia as the central drivers (Hickman et al., 2018). Similar to peripheral immune cells, microglia express surface receptors known as pattern recognition receptors (PRR), which can sense danger- or pathogen-associated molecular patterns (DAMPs or PAMPs). These receptors include Toll-like receptors TLR2, TLR4, and TLR6, as well as cluster of differentiation (CD)36, CD14, Triggering Receptor Expressed on Myeloid Cells 2 (TREM2) and CD33. Notably, TREM2 and CD33 have been identified as AD risk genes through genome-wide association studies (GWAS) (Schwabe et al., 2020). Some PRRs can recognize and bind fibrillary *β*-amyloid (Bamberger et al., 2003; Stewart et al., 2010; Liu et al., 2012). PRR activation triggers the expression of pro-inflammatory cytokines, including interleukin (IL)-1β and IL-18, via the nuclear factor-κB (NF-κB) pathway. These cytokines are produced in an inactive pro-form. The simultaneous activation of the NF-κB pathway and phagocytosis, for example through TREM2 and CD33, as well as lysosomal damage, results in activation of the NLR family pyrin domain containing 3 (NLRP3) inflammasome, a cytosolic multiprotein complex that minimally consists of NLRP3, apoptosis-associated speck-like protein containing a CARD (ASC) and caspase-1. Activation of the NLRP3 inflammasome triggers caspase-1 activation, leading to the maturation of pro-IL1β and pro-IL18 into their active cytokine forms (Heneka et al., 2014). Inflammasome activation also initiates pyroptosis, a lytic regulated cell death mode that releases cytokines into the extracellular environment (Miao et al., 2011).

Microglia can engulf fibrillary Aβ *in vitro*, triggering lysosomal membrane rupture and activation of the NLRP3 activation, which leads to secretion of IL-1β and IL-18 and other pro-inflammatory mediators that promote neuronal death (Halle et al., 2008). The impact of NLRP3 inflammasome activation on amyloid pathology has also been investigated *in vivo* using mouse knockout models. *Nlrp3* inflammasome deficiency in transgenic APP/PS1 mice was shown to reduce Aβ plaque load and significantly alleviate memory loss and pathological behavior (Heneka et al., 2012; Venegas et al., 2017). However, a recent report did not find consistent evidence supporting a broad role for the NLRP3 inflammasome in APP/PS1 mice and other preclinical models of Aβ-induced neuroinflammation (Srinivasan et al., 2024).

NLRP3 activation has also been associated with tau pathology. *In vitro* studies have shown that following uptake by microglia, tau seeds can activate the NLRP3 pathway similarly to Aβ aggregates, by inducing lysosomal destabilization (Ising et al., 2019; Stancu et al., 2019). *In vivo, Nlrp3* deficiency in tau22 transgenic mice was shown to reduce tau phosphorylation and aggregation by modulating the activity of tau kinases and phosphatases (Ising et al., 2019). Additionally, ASC deficiency significantly decreased tau pathology in the PS19 transgenic model and attenuated tau seeding in a tau injection model (Stancu et al., 2019). These observations suggest that NLRP3 is a key inflammasome involved in the progression of tau pathology.

In addition to NLRP3, pyroptosis is emerging as key mechanism that contributes to inflammasome-driven pathology across metabolic, autoimmune, and neurodegenerative diseases. Cleavage of GSDMD by caspases 1 and 11 (in mice), or caspases 1, 4, and 5 (in humans) releases an amino-terminal pore-forming GSDMD domain that inserts in the plasma membrane, where it oligomerizes to induce membrane rupture and leakage of the cytosolic contents (Vande Walle and Lamkanfi, 2016). Hence, GSDMD inhibition has been proposed as a novel therapeutic strategy to prevent inflammasome-driven pathology in several diseases, including neurodegenerative diseases (Voet et al., 2019). GSDMD in peripheral myeloid cells was shown to regulate microglial immune training and neuroinflammation in a mouse model of Parkinson’s disease (PD), and PD symptoms were recently shown to be alleviated by pharmacological GSDMD inhibition (Wang et al., 2023). Other studies suggest GSDMD as a biomarker for AD, demonstrating higher levels of GSDMD protein in cerebrospinal fluid (CSF) from AD patients (Shen et al., 2021).

In this study, we addressed the importance of NLRP3 inflammasome activation and pyroptosis induction for neurodegeneration and pathology in the tau P301S transgenic mouse model. These mice carry a P301S mutation in the *Mapt* gene under control of the murine Thy1 promotor. Homozygous mutant mice start to display neurofibrillary tangles along with neurodegeneration as early as 4 months of age. Using *Nlrp3^−/−^* mice, our results show that NLRP3 signaling is dispensable for tau pathology in P301S mice. Moreover, full-body and microglia-selective *Gsdmd* deficiency failed to ameliorate tau pathology P301S mice. However, we provide evidence that organotypic slice cultures from these mice represent a robust *ex vivo* mechanistic model to evaluate NLRP3 pathway activation and pharmacological inhibitors.

## Materials and methods

### Animals

Thy1-hTau.P301S transgenic mice expressing the microtubule-associated protein tau (MAPT) with the human P301S mutation under control of the Thy1 promoter have previously been described (Allen et al., 2002). Thy1-hTau.P301S mice were crossed with full-body *Nlrp3* knockout mice, purchased from the Jackson Laboratories (B6.129S6-*Nlrp3*^tm1Bhk^/J), to generate *Nlrp3*^+/+^xP301S^Tg/Tg^ and *Nlrp3*^−/−^xP301S^Tg/Tg^ mice (named hereafter *Nlrp3*xP301S).

A conditional *Gsdmd* knockout model was generated in collaboration with Cyagen. In the targeting vector, the Neo cassette was flanked by self-deletion anchor (SDA) sites and used for negative selection. Mouse genomic fragments containing homology arms (HAs) and conditional knockout (cKO) region were amplified from a BAC clone by using high fidelity Taq DNA polymerase and were sequentially assembled into the targeting vector together with recombination sites and selection markers. The *Gsdmd* targeting construct was linearized and transfected into C57Bl/6 embryonic stem (ES) cells according to Cyagen’s electroporation procedures. Targeted ES cell clones were injected into C57Bl/6 albino embryos followed by re-implantation into CD-1 pseudo-pregnant females, and subsequently bred with C57/Bl6 females. Full-body *Gsdmd* knockout mice were generated by crossing the floxed *Gsdmd* line with a Cre deleter line (from Takara) and were further crossed with Thy1-hTau.P301S to obtain *Gsdmd^+/+^*xP301S and *Gsdmd^−/−^*xP301S mice (named hereafter *Gsdmd*xP301S). Both male and female animals were used (approximately the same number of males/females per genotype).

Conditional floxed *Gsdmd* mice were crossed with Cx3Cr1Ert2-Cre transgenic mice (Goldmann et al., 2013) to generate tamoxifen-inducible myeloid-specific *Gsdmd* knockout mice, and further crossed with Thy1-hTau.P301S transgenic mice. *Gsdmd*^FL^ mice, expressing the floxed allele, are used as controls in all experiments. At 4–6 weeks of age, *Gsdmd*^FL^ and *Gsdmd*^Cx3Cr1-KO^ mice were subcutaneously injected with tamoxifen (20 mg/mL, Sigma-Aldrich T5648) dissolved in corn oil (Sigma-Aldrich, C8267) twice, 48 h apart, to activate Cre recombinase. All experiments were performed on mice backcrossed into the C57BL/6 genetic background. Mice were housed in individually ventilated cages in a specific pathogen-free facility at Janssen Pharmaceutica, Beerse, Belgium, or at VIB-UGhent, Belgium. All experiments were conducted according to institutional, national, and European animal regulations. Animal protocols were approved by the ethics committee of Janssen Pharmaceutica, or by the ethics committee of Ghent University.

### Genotyping of the different mouse models

The P301S tau mouse model was genetically validated using pcr protocol. DNA was mixed with 10 μM P1 GATCTTAGCAACGTCCAGTCC (Metabion), 10 μM P2 TGCCTAATGAGCCACACTTG (Metabion), 5 μM probe 5’[6FAM]-TTTGTAGACTATTTGCACACTGCCGC-[BHQ1]-3′ (Metabion) and H_2_O (5Prime). The following pcr conditions were used: 50°C for 120 s, followed by 94°C for 600 s, ending at 40 cycles of 92°C for 30s and 60°C for 60s.

*Nlrp3* or *Gsdmd* deficient P301S mice were also analyzed with pcr. DNA from Nlrp3tm1Bhk (Mut) mice or *Gsdmd* mice was mixed with 2x buffer (Promega), 2 mM MgCl2 (Promega), 10 mM dNTPs (Promega), go Taq M829 (5 U/μl, Promega), H_2_O (5Prime) and 10 μM of each of the following primers, supplied by Metabion; *Nlrp3*: TTCCATTACAGTCACTCCAGATGT and TGCCTGCTCTTTACTGAAGG, *Gsdmd*: TAGTGATACAGTGGGACAAGCCT, GAGGAACAACTCTACACCTAGGAA and AGGTCAGTACCCCCTTGACC. Denaturation, annealing and elongation steps were executed as follows: 94°C for 120 s, 10 cycles 94°C for 30s, 64–60°C for 30s and 72°C for 60s, followed by 25 cycles of 94°C for 30s, 60°C for 30s and 72°C for 60s to finish at72°C for 300 s. Samples were analyzed using capillary electrophoresis assay HT DNA 5 K and assessed based on fragment size.

### Validation of *Gsdmd* model

#### Western blotting

For Western blot analysis, BMDMs were seeded in a 12-well plate at 1 × 10^6^ cells per ml and lysates collected at 2 h post-treatment using cell lysis buffer [20 mM Tris–HCl (pH 7.4), 200 mM NaCl and 1% NP-40] and Laemmli buffer. Protein samples were boiled at 95°C for 10 min and separated by SDS-PAGE followed by transfer to PVDF membranes. Blocking, incubation with antibody, and washing of the membranes were done in PBS supplemented with 0.05% Tween 20 (v/v) and 3% (w/v) non-fat dry milk. Immunoblots were incubated overnight with primary antibodies against *Gsdmd* (#209845, #219800; Abcam). Horseradish peroxidase-conjugated anti-rabbit secondary antibody was used to detect proteins by enhanced chemiluminescence (Pierce, Thermo).

#### Cytokine analysis

Supernatants from stimulated cells was collected at 2 h post-treatment and Luminex assay for IL-18, IL-1β, IL-6 and TNF (EPX01A-10267-901; EPX01A-10224-901; EPX01A-10213-901; EPX01A-10223-901; Procartaplex, Thermo Fisher) was performed.

### Immunohistochemistry (IHC)

One hemisphere of each mouse brain was post-fixed overnight in a formalin-based fixative and embedded in paraffin. Five μM thick sections were collected using a microtome. Two brain sections from each mouse (at two different levels) were used. For this staining, Bond Polymer Refine Detection (Leica, DS9800) was used on the Leica Bond autostainer RX. Following deparaffinization and rehydration of the sections, antigen retrieval was performed with Heat Induced Epitope Retrieval (citrate buffer, pH 6) and endogenous peroxidase activity was blocked with 3% hydrogen peroxide (DAKO, Glostrup, Denmark). Samples were incubated for 1 h with AT100 [0.1 μg/mL, Thermo Scientific, Merelbeke, Belgium (MN1060)] or NeuN primary antibody (1 μg/mL, Millipore, Temecula, United States) diluted in antibody diluent (Leica Bond, AR9352). After extensive washing, sections were incubated with HRP labeled anti-mouse secondary antibody (DAKO, K4001) for 30 min, followed by chromogenic labeling with 3,3-diaminobenzidine (DAB, DAKO). Slides were counterstained with hematoxylin, dehydrated and permanently mounted (Vectamount, Vector Labs, Burlingame, CA, United States). Images were acquired with the Hamamatsu NanoZoomer slide scanner (Hamamatsu Photonics, Shizuoka, Japan) and analyzed using Phaedra. Regions of interest (ROIs) were manually delineated in accordance with the Franklin and Paxinos atlas and for each ROI the DAB-labeled area or number of objects was calculated per total area.

### Neurofilament light chain assay

Mouse plasma was diluted 1/50 and mouse brain stem extracts 1/10000 in NF-Light Sample Diluent (102,252, Quanterix) into a single molecule array (Simoa) HD-1 96 Well HV Plate barcoded (103,022, Quanterix). Simoa NF-Light calibrators and controls (103,186, Quanterix) were added to the 96-well plate undiluted. These samples were diluted by the Simoa HD-X (Quanterix) with a dilution factor of four. The resorufin *β*-D-galactopyranoside substrate (RGB Reagent, 103,159, Quanterix) was heated at 30°C while constantly shaking for 30 min before it was placed into the Simoa reagent bay. The NF-Light Bead Reagent (102,246, Quanterix) was vortexed for 30 s before loading it into the reagent bay. All other reagents from the Simoa NF-Light Reagent Kit (102,186, Quanterix) were placed into the Simoa reagent bay without any prior actions. Subsequently, the 96-well plate was loaded into the Simoa plate rack, and the automated NF-Light assay was started.

The NF-Light assay is a 2-step immunoassay. In a first step, the sample is combined with the NF-Light antibody coated beads and the biotinylated detector antibodies. If NF-Light is present in the sample, the antibody coated beads will bind the target which will also bind to the biotinylated detector antibodies. Following a washing step, streptavidin-*β*-galactosidase will bind to the biotinylated detector antibodies. After another washing step, the beads are resuspended in RBG substrate and transferred onto individual wells in the Simoa Disk (100,001, Quanterix). If NF-Light was captured by the beads, streptavidin beta galactosidase (SBG) will hydrolyze the RBG substrate which will result in a fluorescent signand, together with al and will be detected by the Simoa optical system. The fluorescent signal (at high NF-Light concentrations), and the number of positive wells (at low NF-Light concentrations) are proportional to the NF-Light concentration in the sample. This concentration is interpolated from the calibration curve.

### Tau biochemistry

#### Sarcosyl extraction

Tissue was weighed and homogenized in 2000 μL of buffer H per 100 mg tissue (10 mM Tris, 800 mM NaCl, 1 mM EGTA and 10% sucrose/ pH 7.4). The homogenate was centrifuged at 34000 x g for 20 min and 1% N-lauroylsarcosine was added to the supernatant. After 90 min, the solutions were again centrifuged at 184000 x g for 1 h. The supernatants were kept as sarcosyl-soluble fraction, whereas the pellet containing the sarcosyl-insoluble material was resuspended in homogenization buffer.

#### Mesoscale discovery assay

Coating antibodies (AT8, PT51) were diluted in PBS (1 μg/mL) and aliquoted into MSD plates (30 μL per well) (L15XA, MSD, Rockville, MD, United States), which were incubated overnight at 4°C. After washing with 5 × 200 μL of PBS/0.5%Tween-20, the plates were blocked with 0.1% casein in PBS. After adding samples and standards (both diluted in 0.1% casein in PBS), the plates were incubated overnight at 4°C. Subsequently, plates were washed with 5 × 200 μL of PBS/0.5% Tween-20, and SULFO-TAG™ conjugated detection antibodies (AT8, PT51) in 0.1% casein in PBS were added and incubated for 2 h at room temperature while shaking at 600 rpm. After a final wash (5 × 200 μL of PBS/0.5% Tween-20), 150 μL of 2 x buffer T (MSD) was added, and plates were read with an MSD imager. Raw signals were normalized against a standard curve consisting of 16 dilutions of a sarcosyl-insoluble prep from postmortem AD brain (ePHF or enriched paired helical filaments) and were expressed as arbitrary units (AU) ePHF.

### MAP2 assay

Microtubule-Associated Protein 2 (MAP2) levels were quantitatively measured in brain stem tissue of mice using enzyme-linked immunosorbent assay kit (MyBiosource, MBS2022847). The kit is supplied with pre-coated 96-well strip plates, standard with standard diluent, detection reagent A and B with assay diluent A and B, TMB substrate, stop solution and 30x concentrated wash buffer. The standard was reconstituted in standard diluent starting at 20 ng/mL with 1/2 serial dilution up to eight calibrator points including a blank as 0 ng/mL (diluent). Samples were 1/40 diluted in 1x PBS and, together with the standard, incubated for 1 h at 37°C (100 μL/well). Liquid was removed and no wash was performed before addition of detection reagent A (100 μL/well). After 1 h incubation at 37°C, 1x wash solution was prepared with deionized water and strips were washed three times with 350 μL/well. Next, working solution with detection reagent B was incubated for 30 min at 37°C (100 μL/well). The washing step was repeated followed by TMB substrate incubation for 20 min at 37°C in the dark. After addition of stop solution, strips were read at 450 nm using a microplate reader (SpectraMax^®^ Plus 384 Absorbance Plate Reader, Molecular Devices).

### Protein quantification

To determine protein concentration, we performed Micro BCA Protein Assay Kit (23,235, Thermo Scientific). Brain stem extracts were diluted 1/40 in 1x PBS. Buffer H (composition see above) was used as control and bovine serum albumin as calibrator. After addition of the working reagent with 25 parts of Micro BCA Reagent MA and 24 parts Reagent MB with one part of Reagent MC, plates were incubated at 37°C for 2 h. Absorbances were measured at 652 nm using SpectraMax^®^ Plus 384 Absorbance Plate Reader, Molecular Devices.

### IL-18 mesoscale assay

For IL-18 concentration measurement in plasma samples, a mouse IL-18 DuoSet ELISA kit (R&D Systems, DY7625-05) was optimized for mesoscale (MSD). Multi array 96-well plates (Meso Scale Discovery, L15XA) were coated with 30 μL/well of IL-18 capture antibody (4 μg/mL diluted in 1x PBS, R&D Systems, DY7628-05–844956), which was supplied by the R&D kit. After overnight incubation at 4°C (without shaking), plates were washed with 5 × 200 μL of PBS/0.5% Tween-20 and blocked with 150 μL/well of MSD blocker A kit (Meso Scale Discovery, R93AA-2) for 1 h at room temperature while shaking at 600 rpm. Samples and standard (R&D Systems, DY7625-05 - 844958) were both diluted in 1% MSD blocker A diluent (i.e., 5% MSD blocker A diluted in 1x PBS) and incubated for 2 h at room temperature while shaking at 600 rpm. After another wash step with 5 × 200 μL of PBS/0.5% Tween-20, a premix of biotin labeled IL-18 detection antibody (0.5 μg/mL diluted in 1% MSD blocker A, R&D Systems, DY7625-05–844957) and streptavidin SULFO-tag antibody (0.25 μg/mL diluted in 1% MSD blocker A, Meso Scale Discovery, R32AD-5) was prepared. Then, plates were incubated with the premix for 2 h at room temperature while shaking at 600 rpm followed by washing with 5 × 200 μL of PBS/0.5% Tween-20. Finally, plates were read with 150 μL/well gold read buffer B (Meso Scale Discovery, R60AM-4) by mesoscale reader sector S600. Raw data were interpolated in the standard curve and concentrations were plotted in a graph.

### OSC preparation and stimulation

Preparation of organotypic hippocampal slice cultures was performed with wild-type BL/6 mice, postnatal day 8. Slices were incubated at 37°C (days *in vitro* DIV1-3) and 35°C (DIV4~), 5% CO_2_ for 14 days in serum medium containing MEM (31095–029, Gibco), Hanks’ buffered salt solution (HBSS) (24020–083, Gibco), HEPES (H0887, Sigma), Tris–HCl (15,568,025, Invitrogen), 7.5%M sodium bicarbonate (S5761, Sigma), sodium pyruvate (S8636, Sigma), penicillin/streptavidin (P4333, Sigma) and horse serum (2361953). NLRP3 signaling was primed by treatment with lipopolysaccharides at 1 μg/mL (from *Escherichia coli* O111:B4, L4130, Sigma-Aldrich) for 3 h followed by activation with nigericin at 10 μM (Nigericin sodium salt, N714, Sigma-Aldrich) for 90 min. Then, 15 min prior to nigericin, slices were incubated with different NLRP3 compound concentrations. After LPS treatment, medium was changed from serum to serum-free medium. After treatment, medium from OSCs was collected and IL-1β cytokine levels were measured using a pre-coated MSD kit. For the OSC K18 model, slices were prepared from tau transgenic P301S mice at postnatal day 8 and cultured for 7 days at 37°C (DIV1-3) and 35°C (DIV4~), 5% CO_2_ in medium described above. NLRP3 inhibitor was given 1 h prior to tau seeding (K18, 333 μM). Slices were treated with 1 μM of a NLRP3 small molecule inhibitor [Compound 1; example 49 of WO2020234715A1 (Novartis AG)]. After 24 h incubation, medium was collected and pro-inflammatory cytokine IL-1β concentrations were determined. K18 seeds were prepared by mixture of NH_4_Ac buffer, myc-tau (K18 P301L) and heparin. After incubation for 5 days at 37°C, centrifugation and ultracentrifugation steps were performed. The pellet was washed and resuspended in PBS in order to obtain the appropriate concentration of fibrils. Lastly, the K18 solution was sonicated and stored at −80°C until use.

## Results

### Nigericin and tau seeds induce NLRP3 pathway activation in mouse organotypic slice cultures

**Figure 1:**
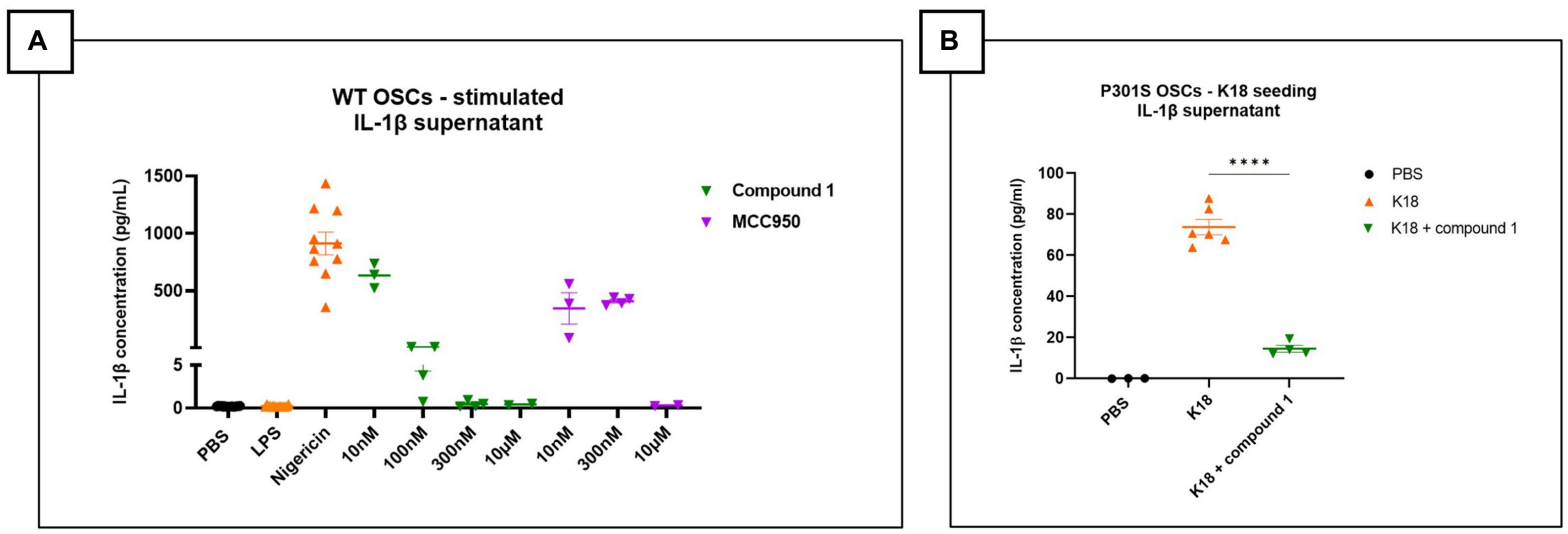
NLRP3 pathway activation in mouse organotypic slice cultures. **(A)** IL-1β measurement in the supernatant of OSCs stimulated with lipopolysaccharides (LPS) and nigericin and treated with NLRP3 small molecule inhibitor (MCC950 or Compound 1). Elevated IL-1β levels in LPS + nigericin condition, which are significantly reduced by increasing concentration of NLRP3 inhibitors. Figure represents two independent experiments combined to increase the replicates per condition. Quantitative data are shown as individual datapoint, mean with standard error. PBS and LPS *n* = 16, Nigericin: *n* = 10, 10 nM, Compound 1: 100 nM (*n* = 3), 300 nM (*n* = 3), 10 μM (*n* = 4); MCC950: 10 nM (*n* = 3), 300 nM (*n* = 4), 10 μM (*n* = 2). **(B)** OSCs supernatant shows elevated IL-1β levels after K18 seeding, which are significantly decreased by addition of 1 μM NLRP3 small molecule inhibitor called JNJ81977038. Data are shown as individual datapoint, mean with standard error. Significance ****p < 0.0001 by one-way ANOVA with multiple comparisons test.

Organotypic slice cultures (OSCs) were derived from C57BL/6 J wild-type mice at postnatal day 8. After 7 days in culture, slices were primed with LPS (1 μg/mL) for 3 h, followed by treatment with increasing concentrations of NLRP3 inhibitors - Compound 1 or the commercially available NLRP3 inhibitor MCC950 - for 15 min before activating the NLRP3 pathway with nigericin (10 μM). Culture medium was collected 90 min post-stimulation to quantify levels of secreted pro-inflammatory cytokines. Elevated IL-1β levels in culture media of OSCs treated with LPS and nigericin were significantly reduced by both NLRP3 inhibitors tested (Figure 1A).

To investigate if tau seeds could activate the NLRP3 pathway, OSCs from Tau-P301S mice were seeded with human tau (K18) P301L mutant pre-formed fibrils only. After a 24 h incubation, secreted IL-1β levels were significantly higher in the K18-seeded OSCs compared to PBS-seeded OSCs. OSCs treated with the small molecule NLRP3 inhibitor Compound 1 1 h before tau fibril seeding showed significantly suppressed K18-induced IL-1β secretion when measured after 24 h incubation (Figure 1B). In conclusion, our findings show significant potential for the use of OSCs as a viable *ex vivo* platform to assess NLRP3 pathway activation and the efficacy of pharmacological NLRP3 inhibitors.

### *Nlrp3* deficiency does not affect tau pathology in P301S mice

The P301S model recapitulates several aspects of human tau pathology, including tau phosphorylation, neurofibrillary tangle formation, neurodegeneration, and motor abnormalities (Allen et al., 2002). To investigate the impact of NLRP3 inhibition on tau pathology *in vivo*, *Nlrp3* knockout mice were crossbred with P301S transgenic mice. Tau pathology in *Nlrp3*^+/+^xP301S^Tg/Tg^ and *Nlrp3*^−/−^xP301S^Tg/Tg^ mice was evaluated histologically at both early (4 months old) and late stages (6 months old) of pathology using the AT100 antibody, which targets the phospho-tau epitopes Thr212 and Ser214. Additionally, biochemical analysis (Mesoscale assay) was performed to measure aggregated tau in the sarcosyl-soluble and insoluble fractions of the brainstem, as well as the total transgenic/human tau in the soluble fraction.

**Figure 2:**
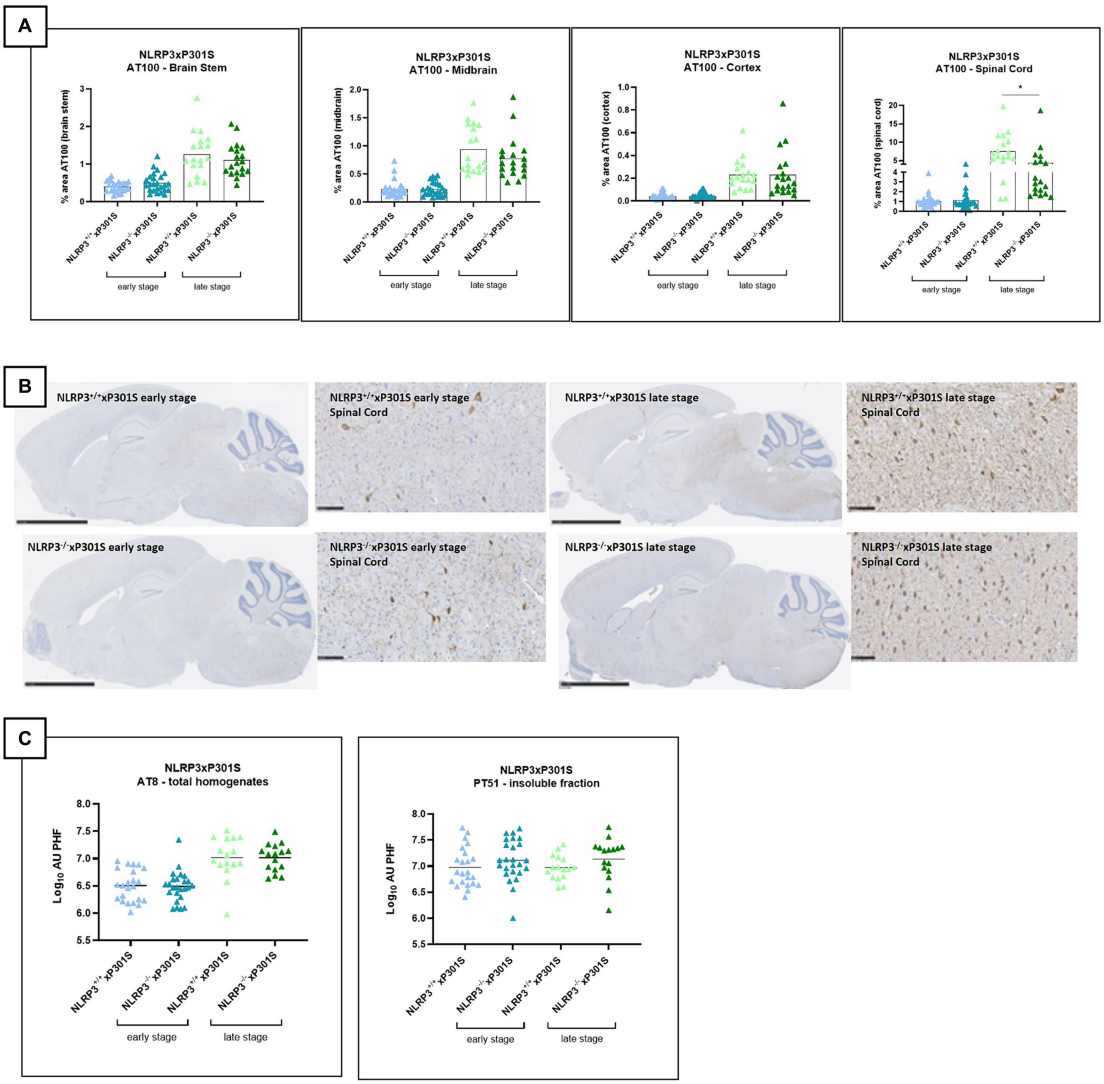
No effect of *Nlrp3* knockout on tau pathology by IHC. **(A)** AT100 immunohistochemistry demonstrated no significant effect on tau phosphorylation/aggregation in all analyzed regions of late-stage *Nlrp3*^−/−^xP301S vs. *Nlrp3*^+/+^xP301S mice. Brain stem, spinal cord, and midbrain showed a trend in reduced pathology for *Nlrp3* deficiency, which was not significant. Early stage P301S mice displayed much lower aggregated tau levels compared to late-stage animals and no effect of *Nlrp3* deletion on tauopathy was observed at this age in any of the regions analyzed. Image analysis data are shown as mean + individual values for each animal (average of two sections/animal). Early stage *Nlrp3*^+/+^xP301S: *n* = 24, *Nlrp3*^−/−^xP301S: *n* = 26; Late-stage *Nlrp3*^+/+^xP301S: *n* = 18, *Nlrp3*^−/−^xP301S: *n* = 19. No significance by unpaired *t*-test of age-matched groups. **(B)** Representative images of the AT00 IHC staining are demonstrated. Scale bars: 2.5 mm (overview images), 100 μm (higher magnification images). **(C)** Biochemical analysis of tau pathology in brainstem of *Nlrp3*xP301S mice. Aggregated tau levels were not significantly reduced in the sarcosyl-insoluble fraction or total homogenate of the brainstem of late-stage *Nlrp3*^−/−^ vs. *Nlrp3*^+/+^xP301S mice. Early stage *Nlrp3*^+/+^xP301S: *n* = 23, *Nlrp3*^−/−^xP301S: *n* = 25; Late-stage *Nlrp3*^+/+^xP301S: *n* = 16, *Nlrp3*^−/−^xP301S: *n* = 16. Data are expressed as mean + individual values of each animal tested (values = average of two sections/mouse).

At the early stage, P301S mice exhibited limited tau hyperphosphorylation, and *Nlrp3* deletion did not affect tau phosphorylation (AT100) in the spinal cord, brainstem, midbrain, and cortex (Figures 2A,B). High levels of phosphorylated/aggregated tau were observed at late stage. However, the late stage is associated with high levels of phosphorylated/aggregated tau. A significant reduction in phosphorylated tau levels was detected only in the spinal cord of *Nlrp3*^−/−^xP301S mice compared to *Nlrp3*^+/+^xP301S mice (Figures 2A,B).

Aggregated and total tau levels in brainstem extracts were measured in both the sarcosyl-insoluble fraction, total homogenate, and soluble fraction. Aggregated tau levels—assessed using PT51/PT51, which is not sensitive to phosphorylation (Vandermeeren et al., 2018) - were unaffected by *Nlrp3* knockout in P301S mice at both early and late stages (Figure 2C). In the total homogenate, aggregated tau levels (analyzed by the AT8/AT8 MSD assay) increased at the late stage of pathology, but *Nlrp3* deficiency did not influence tau pathology (Figure 2C). As expected, total transgenic/human tau levels in the soluble fraction were also not affected by *Nlrp3* deletion in P301S mice (data not shown).

### Microglia-specific *Gsdmd* targeting does not alter tau pathology by IHC or neurofilament levels in P301S mice

To investigate the impact of microglial pyroptosis on tau pathology, we crossed mice carrying floxed *Gsdmd* alleles (*Gsdmd*^FL^) to Cx3cr1CreErt2 knock-in mice to allow Cre recombinase-mediated long-term GSDMD deletion in microglia following tamoxifen (TAM) treatment (Goldmann et al., 2013). Control mice (*Gsdmd*^FL/FL^, expressing GSDMD) and mice with tamoxifen-inducible GSDMD deficiency in microglia (*Gsdmd*^FL/FL^Cx3cr1CreErt2) were then crossed to P301S animals. At 4–6 weeks of age, the progeny was subcutaneously injected with TAM to activate Cre recombinase and generate *Gsdmd*^FL/FL^ P301S mice and *Gsdmd*^FL/FL^Cx3cr1CreErt2 P301S mice that, respectively, do or do not express GSDMD in microglia in P301S mice. *Gsdmd* deletion was evaluated and confirmed *in vitro* using Western blotting and cytokine analysis (Supplementary Figure 2).

**Figure 3:**
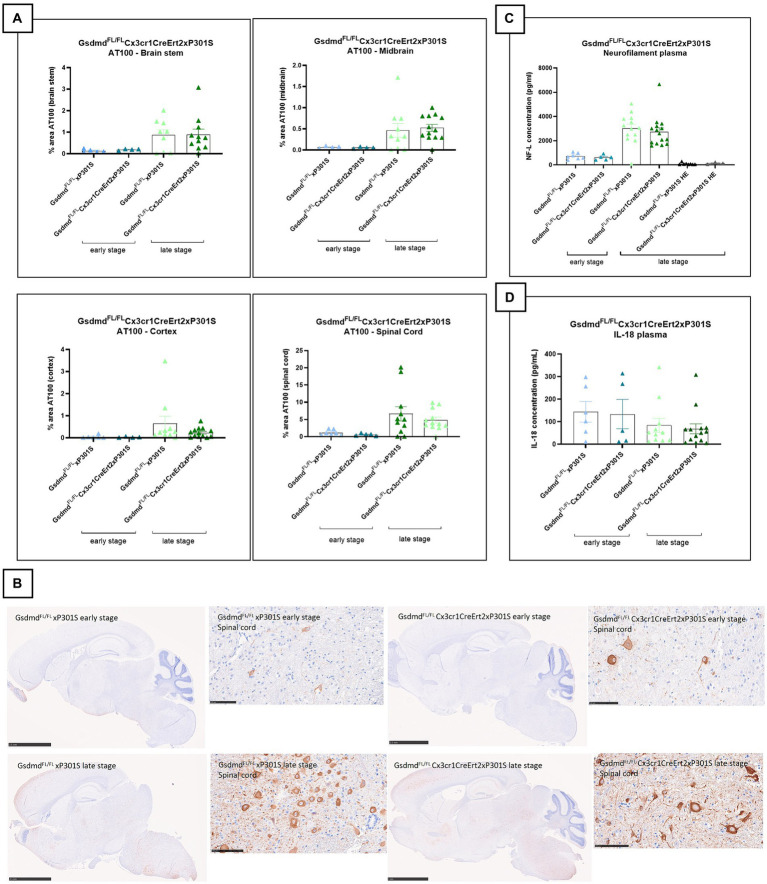
Microglia-specific Gsdmd targeting does not alter tau pathology by IHC or neurofilament levels in P301S mice. **(A)** No significant difference in AT100 levels is detected between *Gsdmd*^FL/FL^xP301S mice and *Gsdmd*^FL/FL^Cx3cr1CreErt2xP301S mice, at early and late stage. Quantitative data are shown as mean + individual values for each animal. Early stage *Gsdmd*^FL/FL^xP301S: *n* = ±6, *Gsdmd*^FL/FL^Cx3cr1CreErt2xP301S: *n* = ±4; Late-stage *Gsdmd*^FL/FL^xP301S: *n* = ±10, *Gsdmd*^FL/FL^Cx3cr1CreErt2xP301S: *n* = ±12. No significance by unpaired *t*-test of age-matched groups. **(B)** Representative images of brain stem, midbrain, spinal cord gray matter, and cortex are shown. The whole brain sections are displayed at 2.5 mm, except for “GsdmdFL/FL Cx3cr1CreErt2xP301S late stage” at 5 mm and the spinal cord sections are displayed at 100 μm. **(C)** Late-stage P301S mice show higher levels of NF-L compared to early stage p301S mice and P301S heterozygous mice. However, no difference in plasma NF-L levels were detected between *Gsdmd*^FL/FL^ xP301S mice and *Gsdmd*^FL/FL^Cx3cr1CreErt2xP301S mice. Early stage *Gsdmd*^FL/FL^ xP301S *n* = 7, *Gsdmd*^FL/FL^Cx3cr1CreErt2xP301S: *n* = 5; Late-stage *Gsdmd*^FL/FL^ xP301S: *n* = 15, *Gsdmd*^FL/FL^Cx3sssscr1CreErt2xP301S: *n* = 14; *Gsdmd*^FL/FL^ xP301S HE: *n* = 6, *Gsdmd*^FL/FL^Cx3cr1CreErt2xP301S HE. No significance by unpaired *t*-test of age-matched groups. **(D)** No differences between *Gsdmd*^FL/FL^ xP301S and *Gsdmd*^FL/FL^Cx3cr1CreErt2xP301S mice on plasma IL-18 cytokine level measured with mesoscale. Early stage Gsdmd^FL/FL^ xP301S *n* = 6, Gsdmd^FL/FL^Cx3cr1CreErt2xP301S: *n* = 5; Late-stage Gsdmd^FL/FL^ xP301S: *n* = 11, Gsdmd^FL/FL^Cx3cr1CreErt2xP301S: *n* = 14. No significance by unpaired *t*-test of age-matched groups.

Brain sections of *Gsdmd*^FL/FL^ P301S and *Gsdmd*^FL/FL^Cx3cr1CreErt2 P301S mice were evaluated for differences in tau pathology and neurodegeneration at early and late stages of the disease. However, no differences in tauopathy or neurodegeneration could be observed between P301S mice that either or not express GSDMD in their microglia (Figures 3A–C). Plasma IL-18 levels were also not affected in P301S mice by microglia-selective *Gsdmd* deletion (Figure 3D). In conclusion, these findings show that microglia-specific *Gsdmd* targeting does not ameliorate tau pathology or neurodegeneration in P301S mice.

### Gasdermin D is dispensable for tau pathology in P301S mice

**Figure 4:**
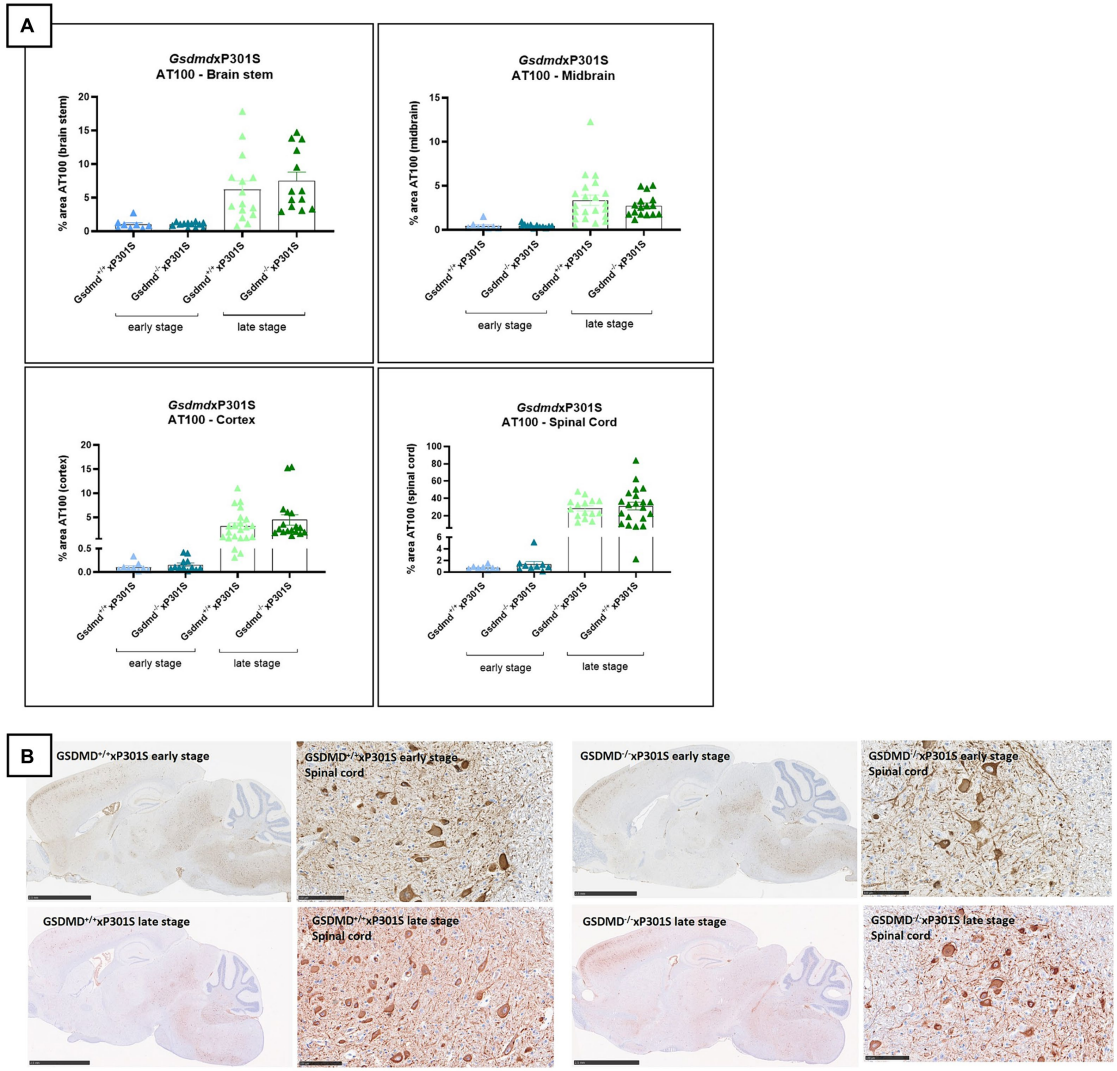
AT100 immunohistochemistry was not altered in *Gsdmd* deficient mice. **(A)** Immunohistochemistry with phospho-tau AT100 antibody showed no significant effect on tau pathology in *Gsdmd*xP301S mice. No significant difference is detected between *Nlrp3*^−/−^xP301S vs. *Nlrp3*^+/+^xP301S mice at early and late stage. Quantitative data are shown as mean + individual values for each animal. Early stage *Nlrp3*^+/+^xP301S: *n* = ±10, *Nlrp3*^−/−^xP301S: *n* = ±15; Late-stage *Nlrp3*^+/+^xP301S: *n* = ±25, *Nlrp3*^−/−^xP301S: *n* = ±20. No significance by unpaired *t*-test of age-matched groups. **(B)** Representative images of brain stem, midbrain, spinal cord, and cortex are shown. The whole brain sections are displayed at 2.5 mm and the spinal cord sections are displayed at 100 μm.

Because the microglia-specific knockout of *Gsdmd* did not yield any results, we were interested to see if full-body the pyroptosis effector protein GSDMD deletion affects tau pathology at all in our read-outs. We therefore crossbred full-body *Gsdmd* knockout mice with P301S transgenic mice. The brains (including brain stem, midbrain, cortex, and spinal cord gray matter) of *Gsdmd*^−/−^xP301S^Tg/Tg^ and *Gsdmd*^+/+^xP301S^Tg/Tg^ mice were analyzed for tau pathology at early and late stages of the disease. However, no significant differences in tau pathology were observed between GSDMD deficient and GSDMD sufficient tau P301S transgenic mice at either age (Figures 4A,B). In conclusion, these findings demonstrate that the pyroptosis effector GSDMD is dispensable for tau pathology in P301S mice.

### Knockout of *Nlrp3*, but not *Gsdmd*, slightly ameliorates neurodegeneration in P301S mice

Tau P301S mice exhibit neuronal loss at a late stage of pathology (Allen et al., 2002). To assess the impact of *Nlrp3* knockout and *Gsdmd* deficiency on neurodegeneration, neurofilament light chain (NF-L) levels were analyzed as a surrogate of neuronal viability. Indeed, NF-L levels in plasma and CSF have been shown to correlate with CNS atrophy/degeneration in human AD and in preclinical models, including the P301S mouse model used in this study (Bacioglu et al., 2016). NF-L was measured using single molecule array (Simoa) immunoassay.

**Figure 5:**
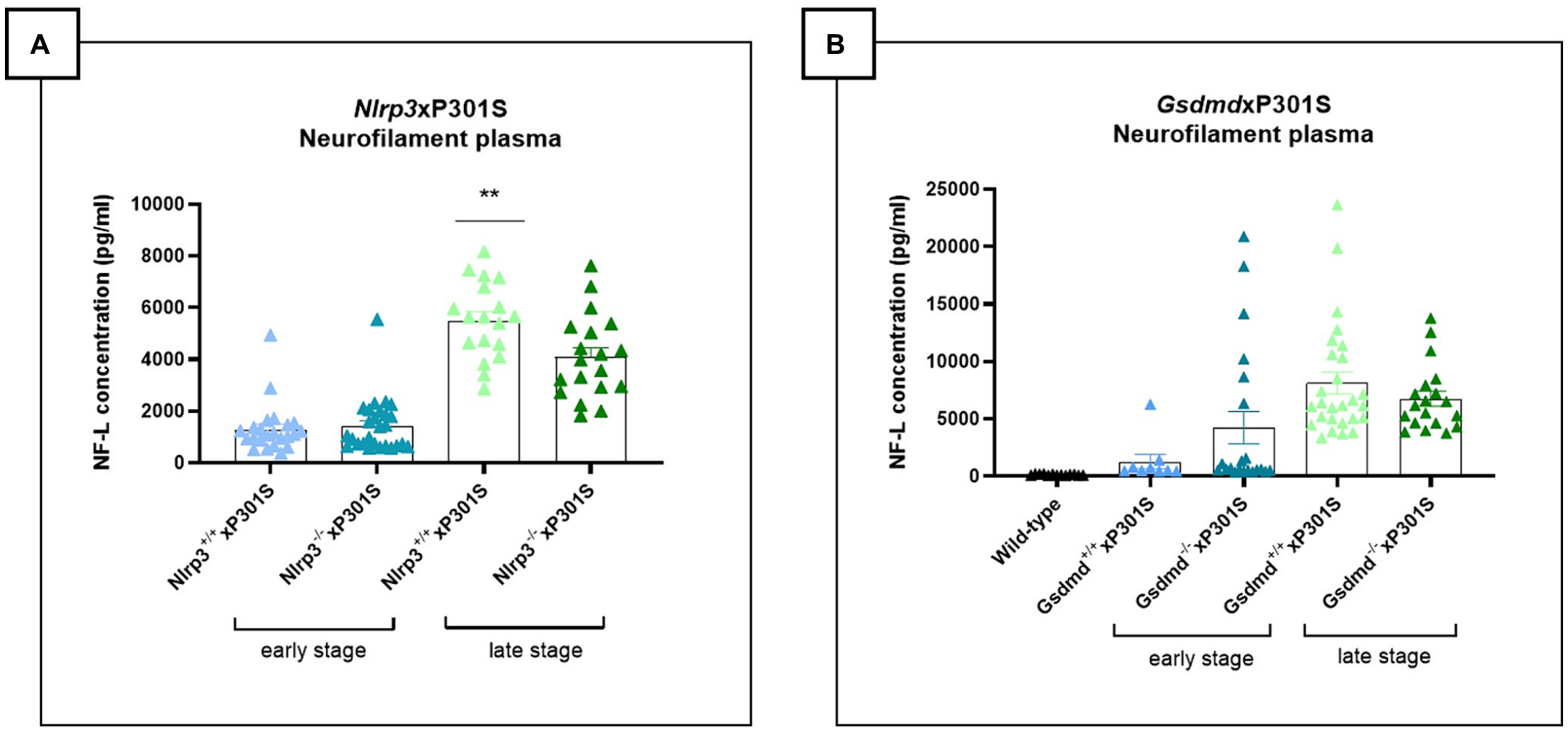
Late-stage *Nlrp3* effect on neurofilament levels and no effect of *Gsdmd* in P301S mice. **(A)** Neurofilament light (NF-L) levels were quantified in plasma of *Nlrp3*xP301S mice as a surrogate marker for neurodegeneration in the CNS. At late stage, NF-L levels were significantly reduced in *Nlrp3* knockout mice, compared to age-related *Nlrp3* WT mice. No effect was demonstrated in early stage P301S mice. Early stage *Nlrp3*^+/+^xP301S: *n* = 23, *Nlrp3*^−/−^xP301S: *n* = 27; late-stage *Nlrp3*^+/+^xP301S: *n* = 18, *Nlrp3*^−/−^xP301S: *n* = 19. **p < 0.01 by paired *t*-test of age-matched groups. **(B)** No effect of GSDMD on plasma NF-L levels were observed in the tau transgenic P301S mouse model. WT: *n* = 17; Early stage *Nlrp3*^+/+^xP301S: *n* = 9, *Nlrp3*^−/−^xP301S: *n* = 21; Late-stage *Nlrp3*^+/+^xP301S: *n* = 27, *Nlrp3*^−/−^xP301S: *n* = 19. No significance by unpaired *t*-test of age-matched groups.

At the late stage of disease, decreased plasma NF-L levels were observed in *Nlrp3* deficient mice compared to wild-type P301S mice (Figure 5A), suggesting a protective effect of NLRP3 deficiency on neurodegeneration in the tau transgenic P301S model. No effect of NLRP3 deletion on neurodegeneration was detected at the early stage of the disease, likely due to the limited neurodegeneration present at this early stage (± 4 months). However, the effect of NLRP3 deletion on plasma NF-L levels at the late stage could not be confirmed in brain stem tissue extracts (Supplementary Figure 1A). Additionally, no differences in levels of microtubule-associated protein 2 (MAP2), another marker of neuronal health, were observed in brain stem homogenates between NLRP3 deficient and sufficient P301S mice (Supplementary Figure 1B).

Plasma and brain stem tissue were also collected from *Gsdmd*^−/−^xP301S and *Gsdmd*^+/+^xP301S mice. At the late stage of the disease, a reduced but not statistically significant trend in NF-L concentration was detected in the plasma of *Gsdmd* deficient mice compared to *Gsdmd* sufficient P301S mice (Figure 5B). In brain stem extracts, however, no differences in NF-L levels could be observed between in *Gsdmd*^−/−^and *Gsdmd*^+/+^P301S mice (Supplementary Figure 1C). Similarly, no differences in MAP2 levels could be shown (Supplementary Figure 1D).

**Figure 6:**
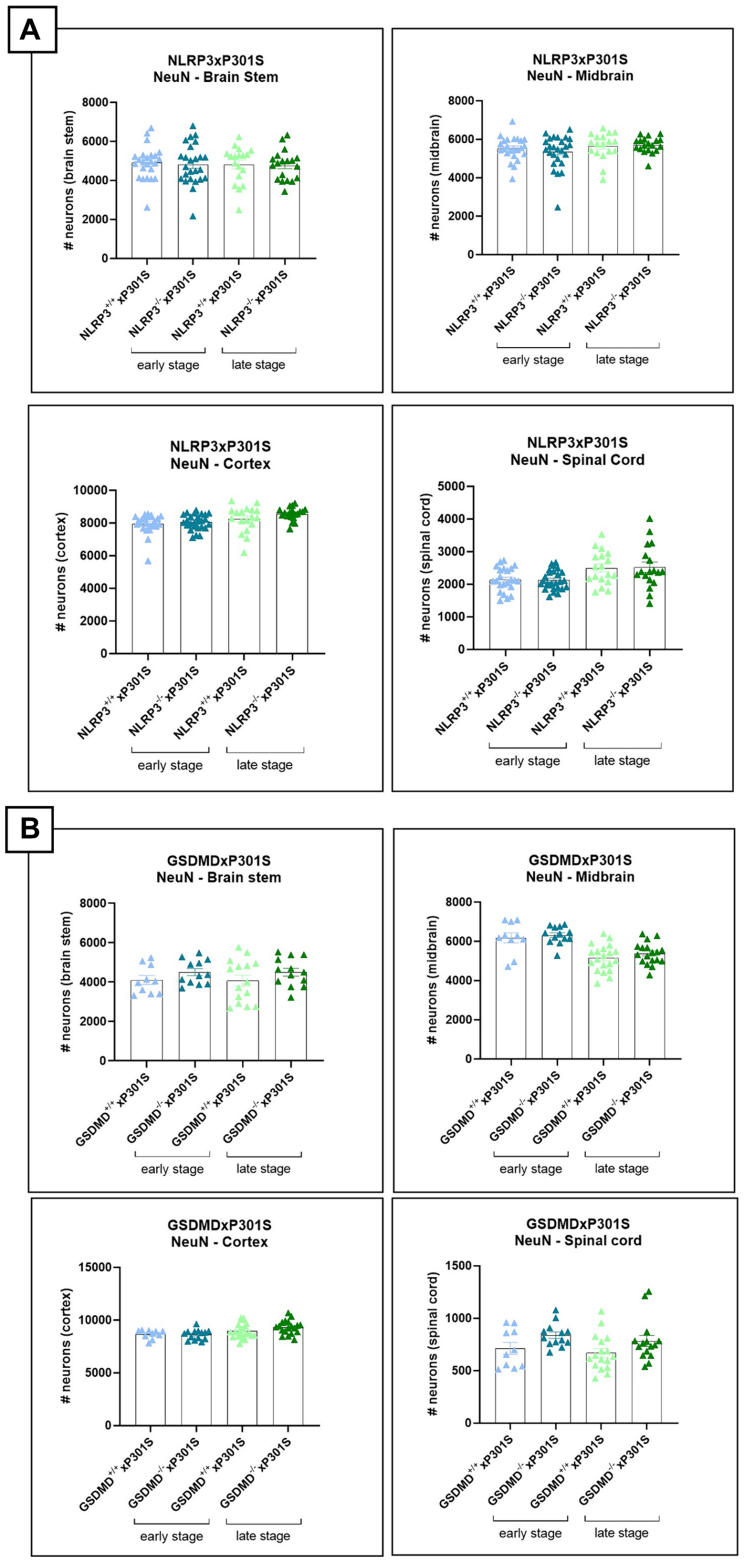
No effect of *Nlrp3* or *Gsdmd* deletion on neuronal count in P301S mice. **(A)** The number of neurons was calculated in brain regions of *Nlrp3*xP301S mice at early and late stage. No effect of Nlrp3 on NeuN levels was demonstrated in the different stages nor the different brain regions of P301S mice. Early stage *Nlrp3*^+/+^xP301S: *n* = ±24, *Nlrp3*^−/−^xP301S: *n* = ±26; late-stage *Nlrp3*^+/+^xP301S: *n* = 18, *Nlrp3*^−/−^xP301S: *n* = ±19. No significance by unpaired *t*-test of age-matched groups. **(B)** Immunohistochemistry with NeuN antibody was also performed on early and late-stage *Gsdmd*^−/−^xP301S *vs. Gsdmd*^+/+^xP301S, demonstrating no effect of GSDMD. Early stage *Gsdmd*^+/+^xP301S: *n* = 10, *Gsdmd*^−/−^xP301S: *n* = ±12; Late-stage *Gsdmd*^+/+^xP301S: *n* = ±20, *Gsdmd*^−/−^xP301S: *n* = ±15. No significance by unpaired *t*-test of age-matched groups.

In addition to NF-L and MAP2 measurements, neurodegeneration was assessed by evaluating the neuronal count in brain slices of mice. Immunohistochemistry was performed using NeuN antibody and demonstrated no difference between *Nlrp3* or *Gsdmd* deficient P301S mice and wild-type P301S mice (Figures 6A,B).

These findings suggest that deficiency in Nlrp3, but not Gsdmd, may slightly alleviate neurodegeneration, in terms of neurofilament levels, in P301S mice. However, further analysis with additional markers of neuronal health is needed to confirm the potential effect of NLRP3 deletion on neuronal health.

### Neither *Gsdmd* nor *Nlrp3* deficiency modulates plasma IL-18 cytokine levels in P301S mice

**Figure 7:**
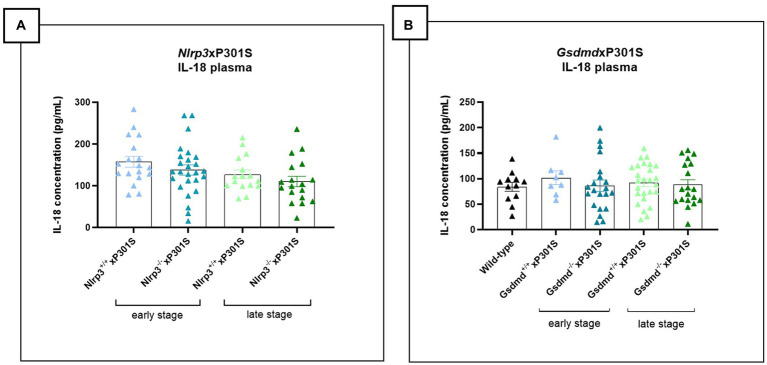
No impact of *Gsdmd* and *Nlrp3* deficiency on plasma IL-18 levels in P301S mice. **(A)** Mesoscale measurement of pro-inflammatory IL-18 levels in plasma of P301S mice. No significant difference between *Nlrp3* knockout mice vs. *Nlrp3* WT mice in early and late-stage P301S mice. Early stage *Nlrp3*^+/+^xP301S: *n* = 18, *Nlrp3*^−/−^xP301S *n* = 26; Late-stage *Nlrp3*^+/+^xP301S: *n* = 16, *Nlrp3*^−/−^xP301S: *n* = 18. No significance by paired *t*-test of age-matched groups. **(B)** IL-18 concentrations were additionally quantified in plasma of early and late-stage *Gsdmd*^−/−^xP301S *vs. Gsdmd*^+/+^xP301S, demonstrating no effect of GSDMD. WT: *n* = 15; Early stage *Nlrp3*^+/+^xP301S: *n* = 8, *Nlrp3*^−/−^xP301S: *n* = 21; Late-stage *Nlrp3*^+/+^xP301S: *n* = 26, *Nlrp3*^−/−^xP301S: *n* = 19. No significance by paired *t*-test of age-matched groups.

Activation of the NLRP3 inflammasome pathway promotes the release of the pro-inflammatory cytokines IL-1β and IL-18. While plasma IL-1β levels were undetectable in P301S mice, plasma IL-18 levels could be measured but did not show differences between wild-type and P301S mice, indicating no significant inflammasome activation. Accordingly, deletion of *Nlrp3* or *Gsdmd* in P301S mice failed to alter plasma IL-18 levels, both at early and late stages of the disease (Figures 7A,B).

## Discussion

It is increasingly recognized that neuroinflammation is not a mere bystander, but an active contributor to the onset and progression of AD. Based on GWAS studies, several immune-linked risk genes have been identified, such as *CD33* and *Trem2*, highlighting the importance of the immune system, and especially of microglia, in AD (Corder et al., 1993; Poirier et al., 1993; Hollingworth et al., 2011; Guerreiro et al., 2013; Jonsson et al., 2013; Benitez et al., 2014; Wightman et al., 2021). However, the role of specific inflammatory pathways and mechanisms of microglial activation in AD-associated neuroinflammation and neurodegeneration is still unclear.

The role of NLRP3 inflammasome activation in different preclinical amyloid models of AD is still unclear (Heneka et al., 2012; Yin et al., 2017; Srinivasan et al., 2024). Furthermore, two recent studies demonstrated the importance of NLRP3 inflammasome activation in promoting tau pathology. NLRP3 and ASC deficiency were shown to reduce tau hyperphosphorylation and aggregation in the Tau22 and PS19 models (Ising et al., 2019; Stancu et al., 2019). Stancu et al. (2019) showed that NLRP3 inhibition and ASC ablation ameliorate tauopathy in PS19 mice, but also revealed a beneficial effect on tau seeding in an injection model, suggesting that NLRP3 activation can aggravate both tau phosphorylation/aggregation and spreading. Other recent work provided mechanistic insights into the mechanisms by which NLRP3 activation regulates the downstream activation or suppression of certain tau kinases and phosphatases, respectively (Ising et al., 2019).

In this study, we investigated the importance of NLRP3 and GSDMD for tau pathology upon genetic ablation of *Nlrp3* or *Gsdmd* in the more aggressive P301S tauopathy model. Our findings demonstrate that *Nlrp3* and *Gsdmd* deficiency in tau P301S mice did not reduce tau phosphorylation and aggregation. In contrast to the Tau22 and PS19 transgenic models used in previous studies (Ising et al., 2019; Stancu et al., 2019), P301S mice show a more aggressive phenotype associated with pronounced neurodegeneration in brainstem and midbrain at the late stage of disease in conjunction with severe tau pathology (Allen et al., 2002). Although *Nlrp3* deficiency slightly reduced plasma neurofilament levels at a late stage of pathology, no effect on tau pathology was observed by IHC and brain stem tissue biochemistry.

In addition to the presented *in vivo* work, we also studied inflammasome activation in the brain parenchyma using the *in vitro* OSC model. Hoyle et al. (2020) previously demonstrated NLRP3 inflammasome activation hippocampal OSCs. We confirmed these findings in our study, demonstrating NLRP3 activation and IL-1β release that could be blocked by pretreatment of OSCs with small molecule NLRP3 inhibitors such as MCC950 and Compound 1. Other studies also demonstrated the potential of OSCs as a robust preclinical model for drug development (Huuskonen et al., 2005). We here further validated the potential of this 3D model for analyzing inflammasome activation in the brain, serving as an interface between cell line studies and *in vivo* models, and have demonstrated its relevance for evaluating NLRP3 pathway activation and pharmacological agents that can inhibit its activation.

## Data Availability

The original contributions presented in the study are included in the article/Supplementary material, further inquiries can be directed to the corresponding author.
