# Supplementary figures and images for: NLRP3 inflammasome activation and pyroptosis are dispensable for tau pathology
Source: Front Aging Neurosci. 2024 Sep 24;16:1459134. doi: 10.3389/fnagi.2024.1459134 (PMC11458539; doi:10.3389/fnagi.2024.1459134)

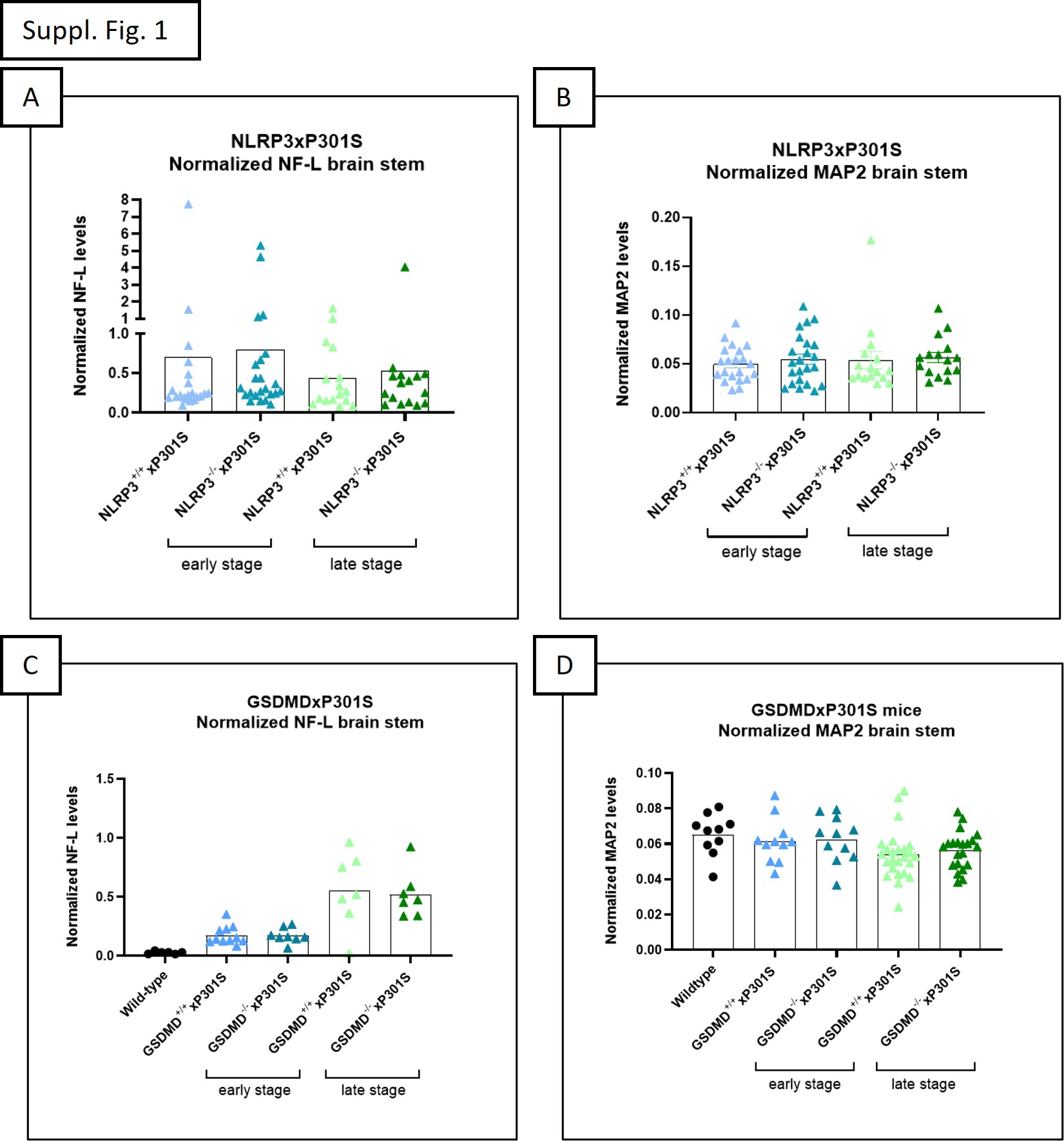

Supplement: Supplementary file 1 [file Image_1.JPEG]

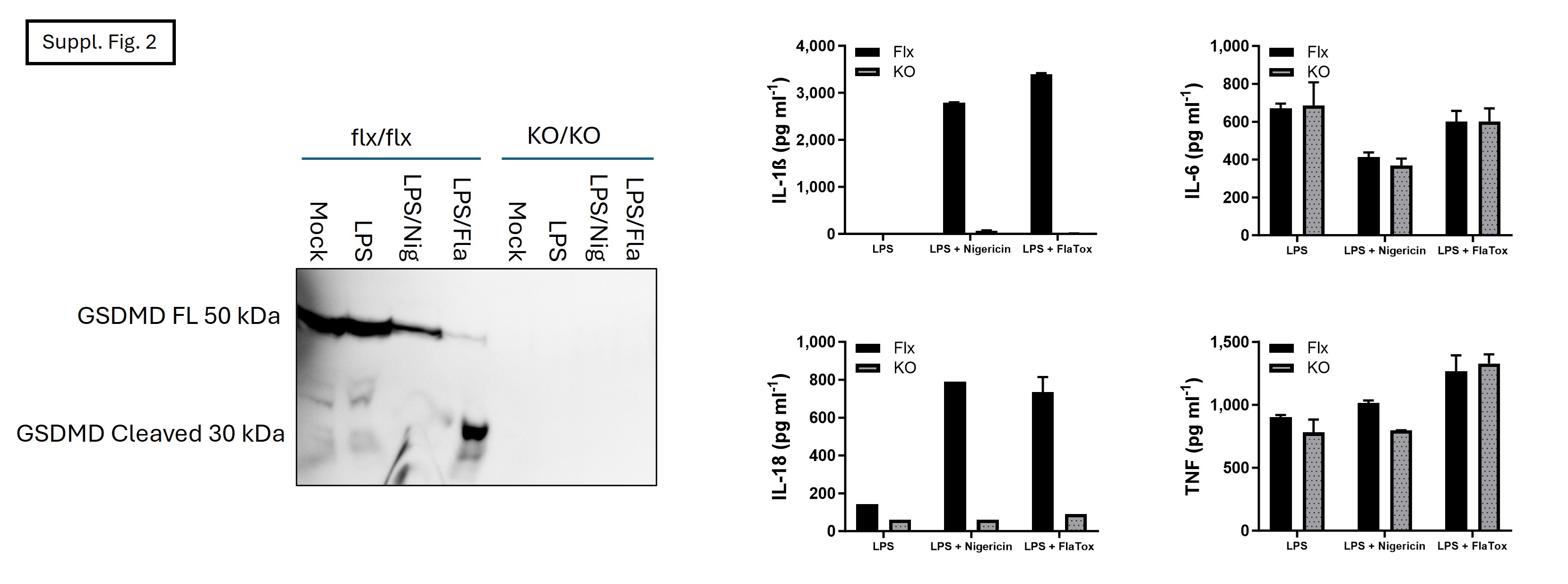

Supplement: Supplementary file 2 [file Image_2.JPEG]
